# Identification of broadly expressed nervous system‐related genes as effective RNAi targets in the brown marmorated stink bug, *Halyomorpha halys*

**DOI:** 10.1002/ps.71021

**Published:** 2026-06-12

**Authors:** Nirakar Panda, Aline Koch, Venkata Partha Sarathi Amineni

**Affiliations:** ^1^ Institute of Phytomedicine, Department of Applied Entomology University of Hohenheim Stuttgart Germany; ^2^ Department of Plant Cell Biology, Biochemistry and Biotechnology, Plant RNA Transport University of Regensburg Regensburg Germany

**Keywords:** dsRNA, dsRNA microinjection, *Halyomorpha halys*, pest management, RNA interference, RNAi target selection

## Abstract

**BACKGROUND:**

The brown marmorated stink bug, *Halyomorpha halys* (BMSB), is an invasive polyphagous pest causing major losses in fruit and field crops in Europe and the Americas. With insecticide options increasingly limited by resistance and regulatory restrictions, RNA interference (RNAi) offers a species‐specific alternative. As RNAi is fully functional in BMSB following hemolymph injection, we evaluated neuronal and neuron‐associated genes as systemic RNAi targets using dsRNA microinjection.

**RESULTS:**

A literature screen of >80 insect RNAi targets identified 12 neuronal/synaptic genes, from which five were selected in BMSB: *acetylcholinesterase* (*HhAChE*), *alpha‐soluble NSF attachment protein* (*HhAsnap*), *Shaker* (*HhSh*), *tyrosine hydroxylase* (*HhTh*) and *Ras opposite protein* (*HhRop*). Previously published transcriptomic data showed *HhAChE, HhSh* and *HhTh* were enriched in brain/central nervous system, whereas *HhAsnap* and *HhRop* were broadly expressed at higher abundance. Adults injected with dsRNA (334–364 bp; 2 μL, 1000 ng/μL) targeting *HhAsnap* or *HhRop* showed strong mortality: 96.8% (30/31) and 100% (34/34) by Day (D)13, respectively (log‐rank, *P* < 0.0001). By contrast, there was no significant difference between insects injected with dsRNA targeting HhAche, HhSh and HhTh and the negative controls. Feeding assays showed >60% reductions in salivary sheath formation from D4 to D8 after dsAsnap or dsRop treatment. Quantitative real‐time (qRT)‐PCR at 72 h confirmed ~58% knockdown of *HhRop*, whereas *HhAChE* and *HhAsnap* showed no significant whole‐body depletion.

**CONCLUSION:**

Broadly and highly expressed targets (*HhAsnap*, *HhRop*) were associated with strong mortality and feeding suppression, whereas brain‐enriched targets did not. Expression breadth and transcript abundance appear critical for RNAi responsiveness in BMSB, identifying *HhAsnap* and *HhRop* as promising candidates for oral or spray‐based RNAi control. © 2026 The Author(s). *Pest Management Science* published by John Wiley & Sons Ltd on behalf of Society of Chemical Industry.

## Graphical abstract

Broadly expressed genes outperformed brain‐enriched neural targets as RNAi candidates in *Halyomorpha halys*. Microinjection of dsRNA targeting *alpha‐soluble NSF attachment protein* (*Asnap*) or *Ras opposite protein* (*Rop*) caused strong feeding suppression and near‐complete mortality, identifying them as promising targets for future oral or spray‐based RNAi pest control.

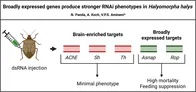

## 1 INTRODUCTION

The brown marmorated stink bug (*Halyomorpha halys*, BMSB), an invasive pest found in Europe and the Americas, poses significant challenges to agricultural management owing to its polyphagous feeding behavior that affects a wide range of host plants, and its high fecundity.^1^, ^2^, ^3^, ^4^ The host range spans crops such as soybean to horticultural species such as tomato, peaches, apples and grapes.^5^ This broad host range complicates population control and often results in significant economic losses, particularly to horticulture. For example, in northern Italy in 2019, the pest is estimated to have caused ≈€588 million worth of damage to fruit production.^6^, ^7^ Within the EU, including in Germany, the use of chemical pesticides is subject to strict regulatory limits: active substances must be approved at EU and national level, and recent strategies aim to halve the use and risk of chemical pesticides by 2030, reducing the set of authorized tools available to growers.^8^ Although the introduction of non‐native parasitoids, such as *Trissolcus japonicus* in Italy, has shown some success in reducing *H. halys* populations, overall effectiveness remains moderate, highlighting the need for new management strategies.^9^

RNA interference (RNAi) is a conserved, sequence‐specific gene‐silencing mechanism present in most eukaryotes, where it regulates gene expression and provides antiviral defense.^10^, ^11^, ^12^ Over the past two decades, RNAi has been developed into a versatile molecular tool, enabling targeted gene knockdown in diverse systems, including mammalian therapeutics and agricultural pest management.^13^, ^14^

In plants and pests, exogenous RNA molecules can be applied as foliar sprays to trigger sequence‐specific silencing, a strategy known as spray‐induced gene silencing (SIGS).^15^ After ingestion or uptake, double‐stranded RNA (dsRNA) is processed by Dicer into short interfering RNAs (siRNAs), which are loaded into the RNA‐induced silencing complex via Argonaut proteins, and guide targeted degradation or translational inhibition of the cognate mRNA.^16^, ^17^, ^18^, ^19^

SIGS has shown effective control of several pests at laboratory scale, including *Leptinotarsa decemlineata, Psylloides chrysocephala, Cylas brunneus, Bemisia tabaci* and *Tetranychus urticae*.^13^, ^17^, ^20^, ^21^, ^22^, ^23^, ^24^ At the commercial level, the first SIGS‐based RNAi insecticide targeting *L. decemlineata* and the acaricide against the honey bee parasite *Varroa destructor* have recently been approved by the U.S. Environmental Protection Agency, demonstrating the translational potential of RNAi‐based products.^25^, ^26^ Nevertheless, RNAi efficacy is highly variable across insect taxa and depends on both the selection of suitable target genes and the efficiency of dsRNA delivery.^13^, ^27^

In BMSB, robust RNAi responses can be achieved when dsRNA is delivered directly into the hemolymph via injection, but oral delivery is markedly less effective, largely owing to strong dsRNase activity in saliva and the gut.^28^ So far, very few studies have found effective RNAi target genes in BMSB and they are broadly active in function, such as *Clathrin heavy chain* (*Chc*), *ATPase N2B, Serine/threonine‐protein phosphatase PP1‐beta catalytic subunit* (*PP1*), *G protein‐coupled receptor 161‐like* (*GPCE*), *Charged multivesicular body protein 4b* (*Snf7*), *Baculoviral IAP repeat‐containing protein 7‐B‐like* (*IAP*) and *Laccase2* (*lac2*).^28^, ^29^, ^30^

Neuronal function in insects is controlled by several key genes that regulate neurotransmission and synaptic activity, including *Acetylcholinesterase* (*AChE*), *alpha‐soluble NSF attachment protein* (*Asnap*), *Ras‐related protein* (*Rop*), *Shaker* (*Sh*) and *tyrosine hydroxylase* (*Th*), to ensure proper signaling.^31^, ^32^, ^33^ *AChE* plays a critical role in terminating neurotransmission at cholinergic synapses by hydrolyzing acetylcholine to acetate and choline‐CoA, thereby preventing overstimulation that could lead to paralysis and eventual death of the insect.^34^ *AChE* also is a target for lethal chemical synthetic insecticides such as organophosphates and carbamates.^35^ The Asnap protein is essential for recycling synaptic vesicles after neurotransmitter release, ensuring rapid availability for subsequent signaling.^32^ The *Sh* gene encodes a critical subunit of voltage‐gated potassium channels, which are essential for cell repolarization and the maintenance of controlled organismal movement.^36^, ^37^ *Th*, also known as tyrosine 3‐monooxygenase, initiates the synthesis of catecholamines such as dopamine, adrenaline and noradrenaline, which are essential for neurotransmission.^33^ *Rop* is essential for the precise docking of synaptic vesicles to the presynaptic membrane, facilitating effective neurotransmitter release.^32^ Targeting these genes with dsRNA has reduced survival in different insect species, for example *AChE* in *B. tabaci* ^38^ and *Helicoverpa armigera*,^34^ *Sh* in *Aedes aegypti*,^37^ *Th* in *Plutella xylostella*,^39^ and *Rop* and *Asnap* in *Tribolium castaneum*.^40^

In this study, we hypothesized that targeting neuronal genes would induce relatively rapid mortality, similar to the fast‐acting effects observed for several neurotoxic synthetic insecticides.^31^ To test this, we selected 2–3 genes in each of two groups: (i) genes with broader expression across multiple tissues including central nervous system (CNS)/brain, i.e. *HhAsnap* and *HhRop*; and (ii) genes with function and expression predominantly enriched in the CNS/brain, i.e. *HhAChE*, *HhTh* and *HhSh*.

By evaluating these targets, our aim was to identify RNAi candidates with the highest potential for effective pest management. This work identifies candidate RNAi targets for further evaluation in delivery systems more relevant to field application, including oral and spray‐based approaches.

## 2 MATERIALS AND METHODS

### 2.1 Literature search for neuronal target gene selection

A comprehensive literature survey was conducted across multiple insect species in which RNAi‐mediated gene silencing had been evaluated, to identify suitable RNAi target genes associated with neuronal function. From >80 reported RNAi targets, 12 genes with well‐established roles in nervous system function were initially identified (see Supporting information Table S1). Five genes, *acetylcholinesterase* (*HhAChE, XM_014435003.1*), *alpha‐soluble NSF attachment protein* (*HhAsnap, XM_014434606.1*), *Shaker* (*HhSh, XM_014415910*), *Tyrosine Hydroxylase* (*HhTh, XM_014435270*) and *Ras Opposite Protein* (*HhRop, XM_014418689*), were selected for experimental validation in *H. halys* based on previously reported RNAi phenotypes, functional relevance in neural signaling and conservation across insect taxa.

### 2.2 Sequence retrieval and phylogenetic analysis

The full‐length mRNA and cDNA sequences corresponding to the selected target genes were retrieved from the *H. halys* genome, which is available in the NCBI database. Gene identity and orthology were confirmed using the OrthoDB database.^41^ Conserved protein domains were identified using the NCBI Conserved Domain Database (CDD).

Multiple sequence alignment and phylogenetic analyses were performed using the www.Phylogeny.fr online platform^42^ with the ‘One Click’ workflow. Predicted *H. halys* protein sequences were compared with homologous insect sequences from GenBank. Alignments were generated with Muscle, curated with gblocks, and phylogenetic trees were constructed using the maximum‐likelihood method in PhyML. Branch support was assessed using the approximate likelihood ratio test (aLRT). Trees were visualized with treedyn. In all cases, *H. halys* sequences clustered with their respective orthologues including *Nezara viridula*, *B. tabaci, S. frugiperda, Spodoptera litura, Bactrocera dorsalis* and *Drosophila melanogaster* (Fig. S1), supporting the identity and evolutionary conservation of the annotated proteins.

Additionally, the identified orthologs are examined for expression in the four organs of adult male *H. halys* including brain/CNS, testis, midgut and salivary glands, using fragments per kilobase of transcript per million mapped reads (FPKM) values generated from RNA‐seq data available from the NCBI Sequence Read Archive (SRA) under the BioProject accession no. PRJNA1451241.^43^

### 2.3 dsRNA synthesis

Primer pairs for dsRNA synthesis were designed from BMSB cDNA sequences using the snapdragon dsRNA design tool (Table S2).^44^ The amplified fragments ranged in size from 334 to 364 bp and added a T7 promoter sequence (5′‐TAATACGACTCACTATAGGG‐3′) at the 5′ end of both the forward and reverse primers. All primers (Table S3) were synthesized by Biomers.net (Germany).

Total RNA was extracted from homogenized adult BMSB using the Monarch® Total RNA Miniprep Kit [New England Biolabs GmbH (NEB), Frankfurt, Germany]. The concentration and quality of the RNA were assessed using a BioPhotometer Plus (Eppendorf, Hamburg, Germany). Complementary DNA (cDNA) was synthesized from 1 μg total RNA using the qScript® cDNA SuperMix Kit (Quantabio, Beverly, MA, USA).

For amplification of dsRNA templates before *in vitro* transcription, PCR reactions (50 μL total volume) were prepared using the ALLin™ Mega HiFi DNA Polymerase system according to the manufacturer's instructions, comprising 10 μL of 5 × ALLin™ Mega HiFi buffer, 2 μL each of forward and reverse primers, 0.5 μL ALLin™ Mega HiFi DNA Polymerase, 1 μL cDNA template (50 ng μL^−1^) and nuclease‐free water adjusted to volume. Amplification was performed on a thermal cycler with an initial denaturation at 95 °C for 1 min, followed by a low‐stringency phase of 5 cycles of 95 °C for 15 s, 56 °C for 15 s and 72 °C for 30 s to facilitate incorporation of the T7 promoter sequences, and then a high‐stringency amplification phase of 30 cycles of 95 °C for 30 s, 65 °C for 30 s and 72 °C for 45 s to enrich target‐specific products, with a final extension at 72 °C for 5 min. The PCR products were then purified using the PCR Clean‐Up System (Wizard® SV; Promega, Walldorf, Germany).

The purified amplicons were then used as templates for *in vitro* transcription using the MEGAscript™ T7 Transcription Kit (Thermo Fisher Scientific, Waltham, MA, USA). Following an incubation period of approximately 14 h at 37 °C, the synthesized dsRNA was treated with DNase‐I (RNase‐free) enzyme (NEB, Germany), purified and eluted in nuclease‐free water using Monarch® Spin RNA Cleanup Kit (μg) (NEB). It was then quantified spectrophotometrically, verified for integrity on 1% agarose gels and stored at −20 °C until use.

### 2.4 dsRNA microinjection and survival bioassays

Adult BMSB insects were reared and maintained as described previously.^28^ Each insect was injected with 2 μL dsRNA (1000 ng μL^−1^) into the ventral abdomen, between the second and third sternites, while under ice anesthesia. Injections were performed using a NanoFil 100‐μL syringe fitted with a 35 G bevel‐cut needle (NF35BV‐2), which was connected to a UMP3 UltraMicroPump (WPI, Sarasota, FL, USA). Nuclease‐free water was injected as a negative control.

Following injection, the insects were kept in groups on fresh green beans and water under control conditions. Mortality was recorded daily for 13 days postinjection (dpi). Individuals that died within 72 h of injection were excluded from subsequent analyses as handling‐related mortality.

In the first experimental replicate, all five candidate genes were evaluated with 17–18 adult BMSBs injected per treatment. Initial screening included all five targets, whereas subsequent replicates focused on *HhAsnap, HhRop* and *HhAChE* based on preliminary phenotypic responses. The number of insects used per treatment across replicates is provided in Table S5. Survival analysis was conducted using the Kaplan–Meier method in prism (v10; GraphPad, Boston, MA, USA). Mortality on 13 dpi was analyzed using a one‐way ANOVA, followed by a Tukey–Kramer honestly significant difference (HSD) *post hoc* test (*P* < 0.05), in jmp pro 18.2 (SAS Institute, Cary, NC, USA).

### 2.5 Feeding site quantification

In order to evaluate the effect of dsRNA treatment on the feeding behavior of adult BMSB, the salivary sheath staining method described previously was used.^28^ Feeding sites on fresh green beans were visualized by immersing the beans in distilled water containing 1–2 drops of blue bromophenol blue based FCF food dye (Dr Oetker GmbH, Bielefeld, Germany) for 5 min. The stained beans were then examined under a stereomicroscope and the feeding sites counted at two‐day intervals for 8 dpi. After each observation, the beans were replaced with fresh ones for subsequent measurements.

Feeding activity was quantified as the average number of feeding sites per insect, calculated by dividing the total number of feeding sites per treatment by the number of surviving insects at each time point. This correction factor compensated for reductions in feeding site counts caused by mortality over time and allowed comparison of feeding rates among treatments with different survival trajectories. The data were analyzed using a standard least‐squares model followed by a Tukey's HSD *post hoc* test in JMP pro 18.2 (SAS Institute).

### 2.6 Quantification of gene silencing by qRT‐PCR

In order to evaluate RNAi‐mediated transcript suppression, adult insects were collected 72 h after dsRNA injection. Total RNA extraction (UV/visible values indicating RNA purity are provided in Table S6) and cDNA synthesis were performed as described above. Quantitative real‐time (qRT)‐PCR was conducted using SYBR Green chemistry (JumpStart Taq ReadyMix; Sigma‐Aldrich/Merck KGaA, Darmstadt, Germany) on a Bio‐Rad CFX96 Real‐Time PCR System.

Primer specificity and amplification efficiency (90–110%; *R* ^2^ > 0.98) were validated using serial cDNA dilutions and melt curve analysis (Table S4). Relative transcript abundance of *Rop*, *AChE* and *Asnap* was calculated using the 2^−ΔΔCt^ method, with previously standardized *18S rRNA* as reference gene.^28^, ^29^ Primer sequences used in this study are provided in Table S3.

Biological replication differed among targets: *HhRop* and *HhAChE* analyses included seven independent biological replicates per treatment group derived from two experimental batches, whereas *HhAsnap* expression was analyzed using three biological replicates from a single batch (Table S7). Each biological replicate was measured in technical triplicate.

Statistical analyses were performed on ΔCt values. Where possible, treatment effects were evaluated using a linear model including batch as a covariate; otherwise, unpaired two‐tailed Welch's *t*‐tests were applied. Data are presented as mean ± SEM of 2^−ΔΔCt^ values, with individual biological replicates shown. Differences were considered statistically significant at *P* < 0.05.

## 3 RESULTS

### 3.1 Identification and phylogenetic validation of selected RNAi target genes in BMSB

A comprehensive review of insect RNAi studies identified 82 genes targeted across multiple insect species. Of these, 12 were associated with neuronal or synaptic processes (see Table S1). Five candidate genes (*HhAsnap, HhRop, HhAChE, HhSh* and *HhTh*) were selected for experimental validation in BMSB based on their functional relevance and the lethal phenotypes reported in other insects.

**Figure 1:**
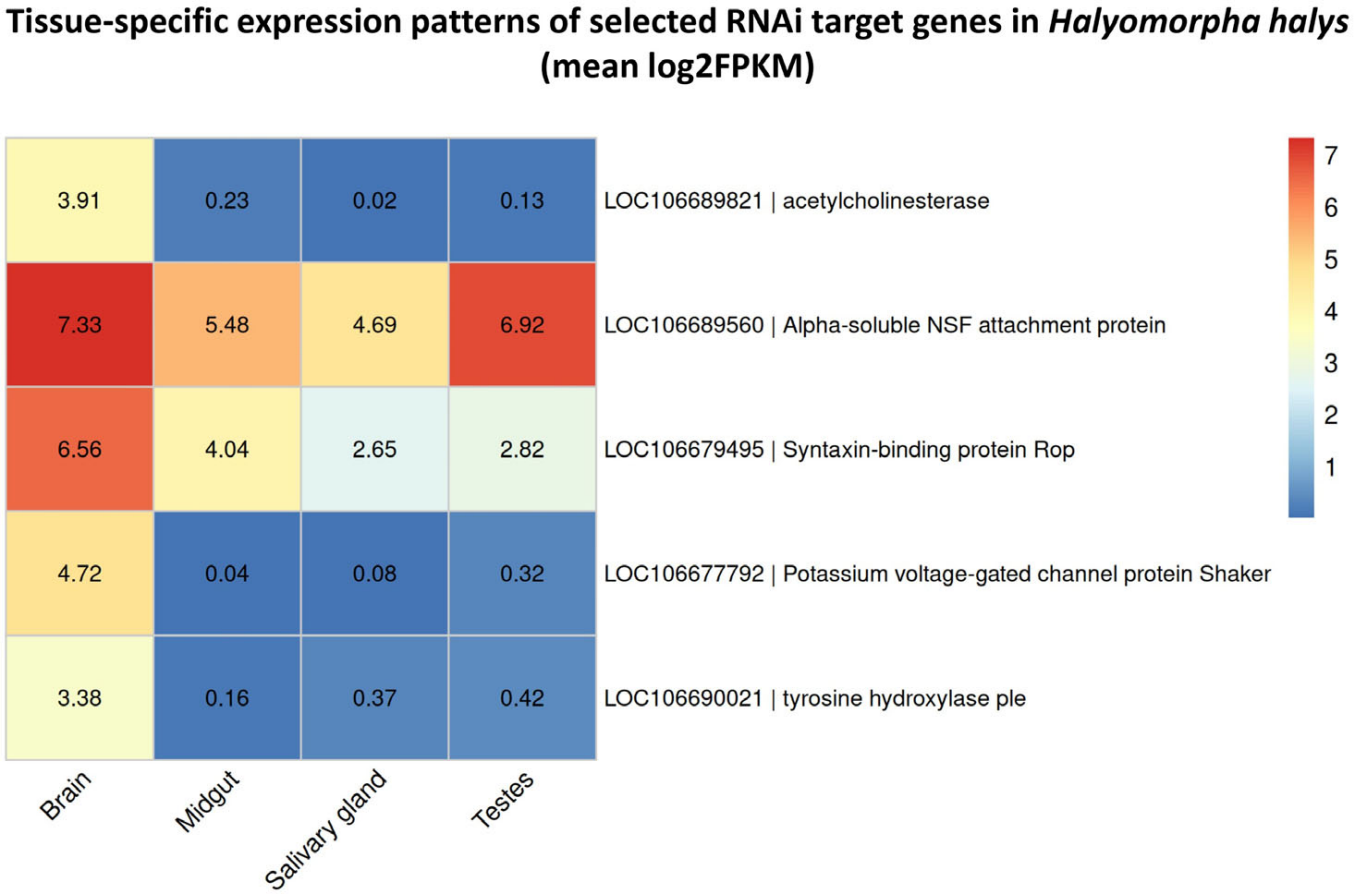
Tissue‐specific expression patterns of selected RNAi target genes in *H. halys*. Heatmap showing mean log₂FPKM for five candidate RNAi target genes across four adult male tissues (brain/CNS, midgut, salivary glands and testes). Expression values represent the average of biological replicates (*n* = 2 for brain; *n* = 3 for other tissues). Rows indicate candidate genes. Color intensity reflects relative transcript abundance, with warmer colors indicating higher expression levels. Numerical values within cells correspond to mean log₂FPKM values. (The raw RNA‐seq files are available in SRA in NCBI under project PRJNA1451241^43^).

Tissue‐specific transcriptomic data from *H. halys* adult male tissues^43^ showed that *HhAChE, HhSh* and *HhTh* were enriched in brain/CNS relative to non‐neural tissues, whereas *HhAsnap* and *HhRop* were expressed more broadly across tissues and at comparatively higher abundance (Fig. 1). Comparative expression resources from *D. melanogaster* (FlyBase^45^) and *T. castaneum* (BeetleAtlas^46^) showed qualitatively similar trends (Figs S2 and S3).

Putative orthologous coding sequences (CDS) for these five targets were identified in the publicly (NCBI) available *H. halys* genome. All dsRNA products designed and synthesized for subsequent bioassays ranged from 334 to 364 bp in length (Table S2). Gel electrophoresis confirmed the quality of the synthesis, displaying single, intact bands without detectable degradation (Fig. S4).

### 3.2 Mortality induced by dsRNA microinjection

In order to evaluate RNAi efficacy, adult BMSB were microinjected with dsRNA targeting the five selected genes or nuclease‐free water. For *HhAsnap*, *HhRop* and *HhAChE*, three independent biological replicates were performed (three independent injection series per treatment), with 31, 34 and 31 insects in total, respectively. For *HhTh* and *HhSh*, a single experimental series was conducted, including 17 and 13 insects, respectively. The negative control group injected with nuclease‐free water comprised three independent series with a total of 34 insects.

**Figure 2:**
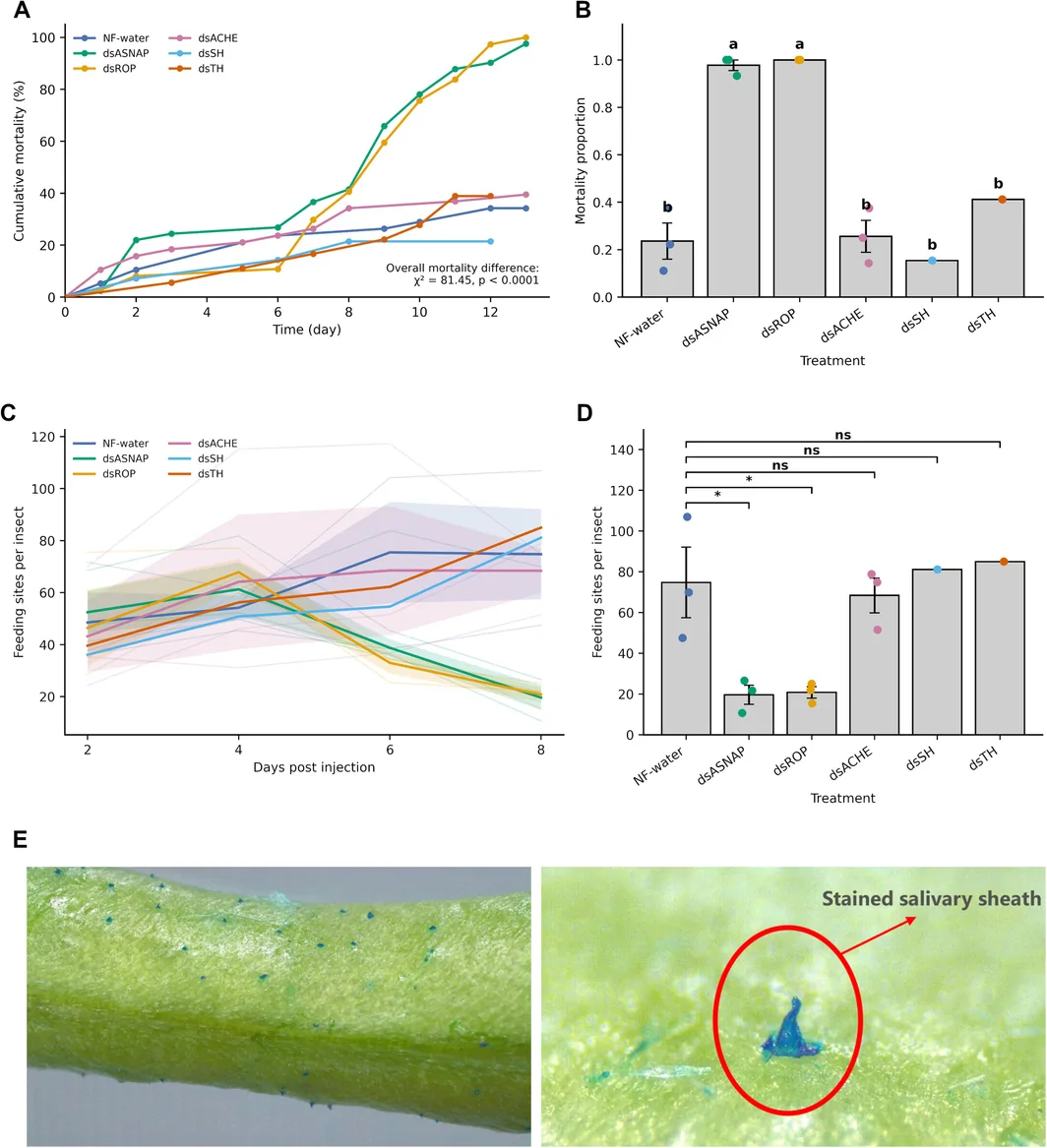
Effects of dsRNA‐mediated gene silencing on survival and feeding behavior in adult *H. halys*. Adult insects were injected with dsRNA targeting dsAsnap, dsRop, dsAChE, dsSh or dsTh, or with nuclease‐free water (NF‐water) as a control. (A) Cumulative mortality (%) over 13 dpi. Survival curves differed significantly among treatments (log‐rank test, *P* < 0.0001). (B) Endpoint mortality proportion. Bars show mean ± SEM with individual replicates. Different letters indicate significant differences (*P* < 0.05). (C) Feeding activity (feeding sites per insect) from 2 to 8 dpi. (D) Feeding sites per insect on 8 dpi. Bars represent SEM with individual replicates; asterisks indicate significant differences; ns, no significant difference. (E) Representative images of salivary sheath staining on green beans. Blue‐stained sheaths indicate feeding sites; the right panel highlights a stained salivary sheath.

Two target genes, *HhAsnap* and *HhRop*, produced strong and consistent lethal phenotypes across all replicates. By 13 dpi, dsRNA‐Asnap (dsAsnap) treatment resulted in 96.8% cumulative mortality (30 of 31 insects), whereas dsRNA‐Rop (dsRop) treatment caused 100% mortality (34 of 34 insects). In both groups, mortality escalated rapidly, exceeding 60% by 9 dpi and 80% by 11 dpi. Kaplan–Meier survival analysis confirmed that survival in dsAsnap‐ and dsRop‐treated insects was significantly lower than in the control group [Mantel–Cox test, *P* < 0.0001, see Fig. 2(A),(B)]. No significant difference was observed between the *HhAsnap* and *HhRop* treatments (Mantel–Cox test, *P* = 0.91).

By contrast, dsRNA treatments targeting *HhAChE* (11 of 31 dead), *HhTh* (seven of 17 dead) *and HhSh* (two of 13 dead) failed to induce significant mortality and were statistically indistinguishable from the negative control group (*P* > 0.05). The control group (injected with nuclease‐free water; nine of 34 dead by 13 dpi) exhibited a background mortality rate of ≈26.5% by 13 dpi. This background mortality likely reflects handling and injection‐associated stress. Consequently, as the *HhAChE*, *HhSh* and *HhTh* treatments did not exceed the baseline injury‐related mortality, under the conditions tested, they were not associated with mortality above the control baseline.

### 3.3 Feeding behavior is strongly suppressed in dsRNA‐treated insects

In order to assess treatment effects on host use beyond survival, the feeding behavior of adult BMSB was monitored for 8 dpi using a salivary sheath staining assay [Fig. 2(E)]. Feeding punctures on stained green beans were counted at 2‐day intervals and converted to mean feeding sites per insect by dividing the total number of sites by the number of surviving insects in each treatment at each time point, allowing separation of changes in feeding rate from changes in survival [see Fig. 2(C),(D)].

Insects injected with *HhAsnap* and *HhRop* dsRNA showed a marked decline in feeding activity over time. For both targets, mean feeding sites per insect decreased steadily between 4 and 8 dpi, with reductions > ~60% relative to earlier time points in several replicate series [Fig. 2(C)]. This decline in feeding coincided with the onset of elevated mortality in these treatments, suggesting that reduced feeding may represent a pre‐lethal impairment associated with progressive physiological dysfunction.

By contrast, insects treated with dsRNA‐AChE (dsAchE) and those in the nuclease‐free water control group generally maintained stable or increasing feeding rates throughout the 8‐day observation period, despite moderate background mortality. Feeding activity in the dsRNA‐Sh (dsSh)‐ and dsRNA‐Th (dsTh)‐treated groups, which were tested in single series, also remained within the range observed for controls, indicating the absence of pronounced pre‐lethal impairment in these treatments.

Statistical analysis of feeding sites per insect on 8 dpi revealed a significant reduction in feeding for dsAsnap‐ and dsRop‐treated insects compared with the nuclease‐free water (*n* = 3, *P* < 0.001). Together with the survival data, these results indicate that RNAi targeting *HhAsnap* and *HhRop* is associated with both increased mortality and a pronounced, temporally coincident suppression of feeding activity, whereas the other dsRNA treatments do not measurably impair feeding behavior.

### 3.4 Quantification of RNAi‐mediated gene silencing by qRT‐PCR

**Figure 3:**
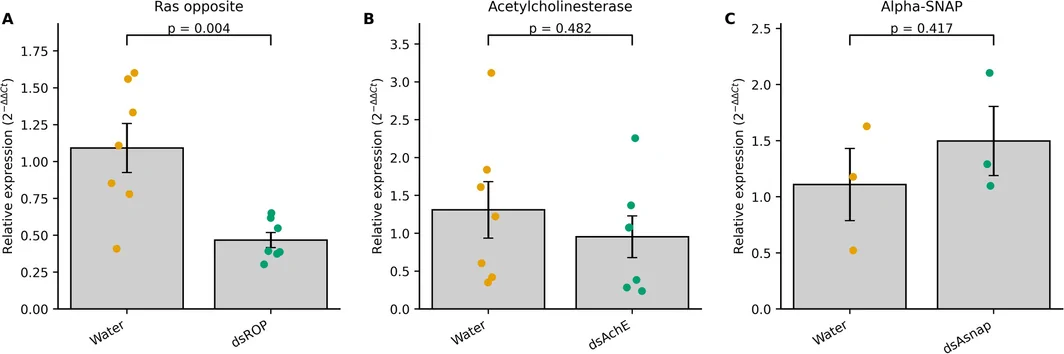
RNAi‐mediated gene silencing assessed by qRT‐PCR. Relative transcript abundance of (A) HhRop, (B) HhAChE and (C) HhAsnap measured 72 hpi of dsRNA. Expression levels were calculated using the 2^−ΔΔCt^ method and normalized to water‐injected controls. Each point represents an individual biological replicate; bars indicate mean ± SEM. Statistical analyses were performed on ΔCt values. For HhRop and HhAChE, treatment effects were evaluated using a linear model including batch as a covariate (*n* = 7 per group). For HhAsnap, comparisons were conducted using an unpaired two‐tailed Welch's *t*‐test (*n* = 3 per group). A significant reduction in HhRop expression was observed following dsRop treatment, whereas no significant differences were detected for HhAChE or HhAsnap. *P*‐values are indicated above each comparison.

Relative transcript levels of *Rop, AChE* and *Asnap* were quantified 72 h after dsRNA injection using qRT‐PCR (Fig. 3).

A significant reduction in *HhROP* transcript abundance was observed in dsRop‐treated insects compared with water‐injected controls (*P* = 0.0037; batch‐adjusted linear model on ΔCt values; see Table S7). Across biological replicates (*n* = 7 per treatment), mean relative expression decreased from ≈1.10 in controls to 0.46 following dsROP treatment, corresponding to an average reduction of ~58%. This reduction was consistent across replicates, indicating effective RNAi‐mediated silencing of *HhROP*.

By contrast, no significant difference in *HhAChE* expression was detected between dsAChE‐treated insects and controls (*P* = 0.482; batch‐adjusted linear model on ΔCt values; see Table S7). Expression levels were highly variable among replicates (*n* = 7 per treatment), and mean expression values were comparable between treatments, indicating that RNAi‐mediated knockdown of *HhAChE* was not consistently achieved under the tested conditions.

Likewise, *alpha‐SNAP* transcript levels were not significantly affected by dsAsnap treatment (*P* = 0.417; Welch's *t*‐test on ΔCt values). Although mean expression appeared slightly higher in dsAsnap‐treated insects compared with controls, this analysis was based on a limited number of biological replicates (*n* = 3 per treatment), and variability among replicates precludes strong conclusions regarding silencing efficiency.

Overall, these results demonstrate gene‐specific RNAi efficiency, with robust and statistically supported knockdown observed for *HhROP*, whereas *HhAChE* and *HhAsnap* did not show significant transcript suppression at 72 h postinjection (hpi).

## 4 DISCUSSION

This study demonstrates that RNAi can be effectively induced in *H. halys* through direct delivery of dsRNA into the hemolymph. However, the efficacy of RNAi strongly depended on the choice of target genes. Silencing the more broadly expressed genes *HhAsnap* and *HhRop* resulted in pronounced phenotypic effects, including significant suppression of feeding behavior from 6 dpi and near‐complete mortality within 13 dpi. By contrast, targeting genes with predominantly brain‐enriched expressions (*HhAChE*, *HhTh* and *HhSh*) did not induce detectable mortality or feeding impairment under the conditions tested. For the subset of genes assessed by qRT‐PCR, whole‐body transcript measurements at 72 hpi also did not show consistent depletion. These findings are consistent with our previous work, in which RNAi targeting of the broadly expressed gene *HhChc* resulted in similarly high mortality following dsRNA injection.^28^

The initial hypothesis of this study was that silencing genes essential for neural signal transmission would result in rapid and severe lethality, analogous to the effects of conventional neurotoxic insecticides.^31^, ^47^ However, contrary to this expectation, dsRNA targeting classical neural‐associated genes (*AChE, Th* and *Sh*) did not produce measurable phenotypic or molecular effects in *H. halys*. This outcome highlights fundamental differences between the modes‐of‐action of chemical neurotoxins and RNAi‐mediated gene silencing, as well as substantial variability in RNAi susceptibility across insect species. Because *HhSh* and *HhTh* were assessed in a single injection series that is lower than *HhAsnap* and *HhRop*, conclusions for these targets should be considered preliminary and warrant confirmation with additional replicates.

Tissue‐specific transcriptomic data from *H. halys* supported a clear distinction between the two target classes tested here: *HhAChE, HhSh* and *HhTh* were enriched in brain tissue, whereas *HhAsnap* and *HhRop* exhibited broader expression across multiple tissues and higher overall transcript abundance (Fig. 1). This species‐specific expression evidence is consistent with the possibility that both expression breadth and transcript abundance may influence RNAi phenotypic outcomes in *H. halys*.

An additional factor that may have influenced RNAi efficacy is the presence of multiple gene isoforms. For several neural‐associated targets, including *HhAChE* and *HhSh*, alternative transcripts or closely related paralogs are known to exist in insects.^48^, ^49^ In such cases, silencing of a single isoform may not be sufficient to disrupt overall gene function if other isoforms can compensate functionally. In the present study, dsRNA constructs were designed against a single annotated transcript per gene, and potential redundancy among isoforms was not explicitly assessed. Therefore, incomplete functional knockdown caused by isoform‐specific targeting cannot be excluded and may have contributed to the lack of observable phenotypic effects for these targets. Future studies incorporating isoform‐aware target design or targeting conserved regions shared across isoforms may help to clarify this aspect.

Strong RNAi‐induced phenotypes following silencing of neural‐associated genes have been reported in several other insect species, indicating that such targets are not universally refractory to RNAi. For example, targeting *HhAChE* has resulted in substantial mortality in *S. exigua*,^50^ *H. armigera* ^34^ and *B. tabaci*,^38^ and targeting *HhSh* has caused high mortality in *A. aegypti* mosquitoes.^37^ However, in contrast to these findings, *H. halys* exhibited no detectable phenotypic or molecular response to *HhAChE* dsRNA, even following direct injection into the hemolymph [Fig. 2(A),(C)].

Several mechanistic factors may explain the limited RNAi phenotypes observed for the more brain‐enriched targets tested here. Although restricted dsRNA or siRNA access to neural tissue is often considered a barrier, recent experiments in the pentatomid *Graphosoma lineatum* and in *L. decemlineata* have demonstrated siRNA localization within CNS tissues after dsRNA injection, suggesting that complete exclusion from the CNS may not be the sole limitation.^51^ However, in the present study, gene silencing was assessed using whole‐body RNA samples, and target‐specific knockdown within brain tissue was not directly evaluated.

Additionally, tissue‐specific expression patterns may have contributed to the observed differences in RNAi efficacy. The more broadly expressed targets (*Asnap* and *HhRop*) showed substantially higher transcript abundance across tissues compared with the more brain‐enriched genes (*AChE*, *Sh* and *Th*; see Fig. 1). Lower baseline expression levels in the latter group may reduce the sensitivity of qRT‐PCR detection and complicate the quantification of knockdown, particularly in whole‐body samples. Furthermore, targeting genes with relatively low and spatially restricted expression may be less likely to induce systemic physiological disruption, especially if effective silencing is not achieved in the relevant tissues or cell types.

Additional factors, including temporal mismatch between transcript measurement and phenotypic onset, protein stability and compensatory transcriptional responses, also may contribute to the observed discrepancies between molecular and phenotypic outcomes.^27^ Consistent with this, qRT‐PCR analysis at 72 hpi did not detect clear transcript depletion for *HhAsnap*, and its transcripts were upregulated. This apparent upregulation may reflect compensatory regulation or sampling variability rather than the absence of RNAi activity. Because only a single time point and whole‐body samples were analyzed, the temporal and tissue‐specific dynamics of gene silencing could not be resolved in this study. In future studies it is important to consider conducting qRT‐PCR at several time points post‐treatment.

By contrast, silencing of *HhAsnap* and *HhRop* resulted in a pronounced decline in feeding activity beginning 6 dpi. The reduction in feeding punctures may reflect impaired physiological function in addition to reduced survival. Although the exact mechanism remains unresolved, these effects may be consistent with broader disruption of cellular processes, potentially involving vesicle trafficking and secretory functions across multiple tissues.

From an applied perspective, the limited efficacy of RNAi in *H. halys* under oral delivery conditions is often attributed to rapid dsRNA degradation in saliva.^28^ However, native RNAi machinery is functional in this species.^28^, ^29^, ^52^ Therefore, improving delivery strategies remains a key challenge. Recent advances in nanoparticle‐based delivery systems and plant‐mediated dsRNA transport highlight promising avenues for enhancing oral RNAi efficacy in sap‐sucking insects.^23^, ^53^, ^54^, ^55^ In this context, the identification of effective targets such as *HhAsnap* and *HhRop* serves as valuable target gene candidates for future oral dsRNA‐delivery optimization.

Taken together, our results suggest that among the neuronal‐associated genes tested, broadly and abundantly expressed targets were more likely to produce strong RNAi‐induced phenotypes than brain‐enriched genes in *H. halys*. These findings support an empirical, phenotype‐driven approach to RNAi target selection, in which expression breadth, transcript abundance and tissue accessibility are evaluated alongside gene essentiality. This interpretation is consistent with genome‐wide RNAi screening in *T. castaneum*, where the most effective targets were enriched in basic cellular processes rather than classical neurotoxic pathways.^27^, ^56^ *HhAsnap* and *HhRop* represent promising candidate targets for future development of RNAi‐based control strategies against *H. halys* and merit further evaluation under realistic delivery scenarios, and in combination with formulation technologies designed to overcome oral delivery barriers.

## AUTHOR CONTRIBUTIONS

Conceptualization, V.P.S.A and A.K.; methodology, V.P.S.A.; software, V.P.S.A.; validation, V.P.S.A, N.P. and A.K.; formal analysis, V.P.S.A. and N.P.; investigation, N.P. and V.P.S.A.; resources, A.K.; data curation, V.P.S.A; writing—original draft preparation, V.P.S.A, and A.K.; writing—review and editing, A.K, N.P. and V.P.S.A.; visualization, V.P.S.A. and N.P.; supervision, V.P.S.A. and A.K.; project administration, V.P.S.A. and A.K.; funding acquisition, A.K. All authors have read and agreed to the published version of the manuscript.

## CONFLICT OF INTEREST

The authors declare no conflicts of interest. The funders had no role in the design of the study; in the collection, analyses, or interpretation of data; in the writing of the manuscript; or in the decision to publish the results.

## Supporting information


**Table S1.** Nervous system‐related RNAi target genes identified from previous insect studies and used for candidate selection in this study.
**Table S2.** Target regions used for dsRNA synthesis against selected *Halyomorpha halys* genes.
**Table S3.** Primer sequences used for dsRNA synthesis and qRT‐PCR analyses.
**Table S4.** Amplification efficiencies and melting temperatures of qRT‐PCR primer pairs used in this study.
**Table S5.** Total number of adult *Halyomorpha halys* insects used in each experimental cohort and treatment group.
**Table S6.** RNA concentration and spectrophotometric purity measurements of RNA samples used for cDNA synthesis and qRT‐PCR analyses.
**Table S7.** RT‐qPCR data used for relative transcript abundance analysis following dsRNA treatment in *Halyomorpha halys*.
**Figure S1.** Maximum‐likelihood phylogenetic analysis of candidate RNAi target proteins identified in *Halyomorpha halys*.
**Figure S2.** Tissue‐specific expression summary of selected candidate genes in *Drosophila melanogaster* based on curated FlyBase expression data (FB2025_05).
**Figure S3.** Adult tissue‐specific expression patterns of selected candidate genes in *Tribolium castaneum* based on BeetleAtlas RNA‐seq data.
**Figure S4.** Agarose gel electrophoresis analysis of purified dsRNA products synthesized for RNAi experiments.

## Data Availability

The data that supports the findings of this study are available in the supplementary material of this article.
