# Supplementary material for: Identification of broadly expressed nervous system‐related genes as effective RNAi targets in the brown marmorated stink bug, Halyomorpha halys
Source: Pest Manag Sci. 2026 Jun 12;82(10):9870–8. doi: 10.1002/ps.71021 (PMC13569117; doi:10.1002/ps.71021)
Supplement: Supplementary file 1 [file PS-82-9870-s001.pdf]

Supplementary Information

Identification of broadly expressed nervous system-related genes as effective RNAi targets in the brown marmorated stink bug, *Halyomorpha halys*

Nirakar Panda,[a,c] Aline Koch[b] and Venkata Partha Sarathi Amineni[a*]

[a] Institute of Phytomedicine, Department of Applied Entomology, University of Hohenheim, Otto-Sander-Straße 5, 70599 Stuttgart, Baden-Württemberg, Germany

[b] Department of Plant Cell Biology, Biochemistry and Biotechnology, Plant RNA Transport, University of Regensburg, Universitätsstraße 31, 93053 Regensburg, Bayern, Germany

[c] Present address: Department of Molecular Genetics, Institute of Biology, University of Hohenheim, Garbenstr. 30, 70599 Stuttgart, Germany

*Correspondence: VPSA: venkata.amineni@uni-hohenheim.de

Table of Contents

Table S1 Nervous system-related RNAi target genes identified from previous insect studies and used for candidate selection in this study. 2
Table S2 Target regions used for dsRNA synthesis against selected Halyomorpha halys genes. 3
Table S3 Primer sequences used for dsRNA synthesis and quantitative RT-PCR analyses. 4
Table S4 Amplification efficiencies and melting temperatures of qRT-PCR primer pairs used in this study. 4
Table S5 Total number of adult Halyomorpha halys insects used in each experimental cohort and treatment group. 5
Table S6 RNA concentration and spectrophotometric purity measurements of RNA samples used for cDNA synthesis and qRT-PCR analyses 6
Table S7 RT-qPCR data used for relative transcript abundance analysis following dsRNA treatment in Halyomorpha halys 7

Figure S1 Maximum-likelihood phylogenetic analysis of candidate RNAi target proteins identified in Halyomorpha halys 8
Figure S2 Tissue-specific expression summary of selected candidate genes in Drosophila melanogaster based on curated FlyBase expression data (FB2025_05). 9
Figure S3 Adult tissue-specific expression patterns of selected candidate genes in Tribolium castaneum based on BeetleAtlas RNA-seq data. 9
Figure S4 Agarose gel electrophoresis analysis of purified dsRNA products synthesized for RNAi experiments. 10

Table S1 ***Nervous system-related RNAi target genes identified from previous insect studies and used for candidate selection in this study.***

The table summarizes previously reported neuronal or synaptic target genes evaluated by RNAi in different insect species, including their proposed biological functions, developmental stage tested, reported mortality, and time required to observe lethal phenotypes. These data were used to guide the selection of candidate genes for experimental validation in Halyomorpha halys. Genes highlighted in grey were selected for experimental validation in this study.

Insect name	Order	Gene	Gene function (simplified)	Survival (days)	Mortality (%)	Growth stage	Reference
Bemisia tabaci	Hemiptera	*Acetylcholinesterase*	Breaks down acetylcholine in synapse	5	100	Adult	(Malik et al., 2016)
Tribolium castaneum	Coleoptera	*Alpha SNAP*	Involved in SNARE complex and synaptic processes	8	100	Larvae	(Ulrich et al., 2015)
Drosophila sp.	Diptera	*Glutamate transporter*	Regulates glutamate; defects cause neurological issues	10–13	100	Adult	(Rival et al., 2004)
Tribolium castaneum	Coleoptera	*Glial cell missing 2*	Controls glial cell development	14	100	Larvae	(Knorr et al., 2018)
Tribolium castaneum	Coleoptera	*Ras opposite*	Involved in neuronal development and vesicle trafficking	8	100	Larvae	(Ulrich et al., 2015)
Aedes aegypti	Diptera	*Ataxin2-binding protein*	Neuro-muscular development	6	87	Adult female	(Mysore et al., 2021)
Aphis gossypii	Hemiptera	*Synapsin*	Regulates neurotransmitter release	4	93	3rd instar	(Qu et al., 2022)
Aedes aegypti	Diptera	*Shaker*	Voltage-gated K^+ channel	6	90	Adult	(Mysore et al., 2020)
Plutella xylostella	Lepidoptera	*Tyrosine hydroxylase*	DOPA precursor;	5	90	NA	(Ellango et al., 2018)
Nilaparvata lugens	Hemiptera	*Neuronal calcium sensor 2*	Regulates Ca^{2+} signaling and neuronal functions	14	80	Nymph (2nd–5th)	(Zhang et al., 2022)
Myzus persicae	Hemiptera	*Voltage-gated sodium channel*	Essential for nerve impulse transmission	7	65.7	3rd instar	(Tariq et al., 2019)
Agrilus planipennis	Coleoptera	*Shibire (Dynamin)*	Involved in endocytosis and vesicle trafficking	8	80	Larvae	(Rodrigues et al., 2018)

Table S2 ***Target regions used for dsRNA synthesis against selected Halyomorpha halys genes.***

Sequences correspond to the amplified coding regions used for in vitro synthesis of dsRNA targeting AChE, Asnap, Rop, Sh, and Th. Transcript accession numbers and amplicon lengths are indicated for each target gene.

dsAChE<br>XM_014435003.1<br>342 bp	CACTCGGGAACATCCACTTTATTATGTATTCAATGCAGAAACAAGCGGTACTGG<br>ACTAGGTCCACGTGCAACAGCATGCGCTTTTTGGAATGAATTCATGCCAAAAC<br>TTTTCCAAAATCCAGATGATAATATTGAATGTAATCAAGGTAATGGAAAACCAC<br>CAAACTATACTATGAGCAGTACCGCAATCCCTCAAGTCATAGAAAGCGGATCTG<br>ACAGATTTCAAATAGCCTGGAGCTGTATAACAGTCTCTCTAATCCTCATCGTTTA<br>TGTAAAATGAGTAGATTCAGGGATCCTTAGTGACGAGCACAGCACGCATAGGG<br>CCTAGAGTAGCCGATGCAC
dsAsnap<br>XM_014434606.1<br>334 bp	CAGAGGCCGAAAAGAAGTTGACTTCCAATAAAAGTTTCTTTGGTTCGCTTTTTG<br>GAGGTACTTCATCAAAAGTTGAAGAAGCTATTGAATGTTATCAAAGGGCAGCAA<br>ATATGTTTAAAATGGCCAAAAAATGGGGAGCTGCAGGAAATGCATTTTGTGAAG<br>CTGCAAATCTCTATTCAAAATTAGGAAGTAGACATGATGCTGCTACTAATTATGTT<br>GATGCAGCAAATTGTTATAAAAAATCTGATCCTAATGAGGGTGTGAATTGCCTTC<br>TGAAGGCTATTGAAATATACACTGACATGGGAAGGTTTACAATGGCTGCAAAACA<br>TCATCA
dsRop<br>XM_014418689<br>340 bp	GTGTCATGCGAGAATCGCTAGGTTTATAAAGACTTTGAAGGAGATAAATATAGCAT<br>TTATTCCGTATGAGCAGCAGGTGTTCTCGCTGGATGCTCCTGAAACGTTCCAGTGC<br>TTCTACAACCCAACATTGAGTAATAGCAGACTTGCAAATATGGAACGTATTGCAGA<br>ACAAATAGCCACATTGTGTGCCACGCTTGGTGAATATCCATCTGTCAGATACAGGA<br>GTGATTTTGATAAAAATGTAGAATTAGCTCAGATAGTGCAGCAGAAATTAGATGCC<br>TACAAAGCTGACGAACCTACAATGGGCGAAGGACCTGAGAAGTCTCGGTCCCAAT<br>TGTTG
dsShaker<br>XM_014415910<br>364 bp	AGTGGGATATGGGGACATGACGCCGGTCGGGGTCTGGGGAAAGATAGTCGGATC<br>ACTGTGCGCCATTGCAGGAGTTCTGACCATCGCCCTTCCCGTGCCCGTCATTGTCTC<br>AAACTTCAACTACTTCTACCACAGGGAGACAGATCAAGAGGATATGCAGTCACAGA<br>ACTTCAATCATGTGACAAGCTGCCCATACCTGCCCAATACACTAGGTCAGCATATGA<br>AGAAAAGTTCGATGTCAGAATCGTCGTCAGACATCATGGAGCTTGAGGAGGGTCT<br>CCTCCTTTCCAGGGATATTGTGAAGAAGCACAATTCGAACCCAAGGCAGCACAATA<br>ACACCATGAGCATCGAAACTGACGTCTGA
dsTh<br>XM_014435270<br>334 bp	TCATGACCAAATACGAGCCAGAATTGGACATGAATCATCCTGGATTTGCTGACAAA<br>GTATACAGGGAAAGGCGAAGGGTCATCGCCAACATTGCTTTCGACTATAAATATGG<br>TGACCCAATCCCAAAGATCGATTACACCCCAGAGGAGACCTCAACCTGGACAGCT<br>GTCTTCAACACTGTCATGGAACTGATGCCAAAGCACTTCTGCAAGGAATACCAACT<br>AGCTTTTTCCAAACTTCAGAATGATGGAATCTTCCGTCCTGACAAAATCCCACAATT<br>AGAAGATATGTCTGCCTTCCTCAAGAAGCACACTGGATTTACCCTAAGGCCTGC

Table S3 ***Primer sequences used for dsRNA synthesis and quantitative RT-PCR analyses.***

T7 promoter sequences were added to dsRNA synthesis primers for in vitro transcription. qRT-PCR primers were designed for quantification of transcript abundance following dsRNA treatment.

Purpose – name	Type	Sequence
dsRNA prep. - dsAChE	Forward	TAATACGACTCACTATAGGGCACTCGGGAACATCCACTTT
dsRNA prep. - dsAChE	Reverse	TAATACGACTCACTATAGGGGTGCATCGGCTACTCTAGGC
dsRNA prep. - dsAsnap	Forward	TAATACGACTCACTATAGGGCAGAGGCCGAAAAGAAGTTG
dsRNA prep. - dsAsnap	Reverse	TAATACGACTCACTATAGGGTGATGATGTTTTGCAGCCAT
dsRNA prep. – dsRop	Forward	TAATACGACTCACTATAGGGGTGTCATGCGAGAATCGCTA
dsRNA prep. – dsRop	Reverse	TAATACGACTCACTATAGGGCAACAATTGGGACCGAGACT
dsRNA prep. - dsShaker	Forward	TAATACGACTCACTATAGGGAGTGGGATATGGGGACATGA
dsRNA prep. - dsShaker	Reverse	TAATACGACTCACTATAGGGTCAGACGTCAGTTTCGATGC
dsRNA prep. - dsTh	Forward	TAATACGACTCACTATAGGGTCATGACCAAATACGAGCCA
dsRNA prep. - dsTh	Reverse	TAATACGACTCACTATAGGGGCAGGCCTTAGGGTAAATCC
qPCR - AchE	Forward	GAACGCGAAGCCATCATATTTC
qPCR - AchE	Reverse	GTCACCAACTACATCAGCTATCA
qPCR - Asnap	Forward	GCCTCTTCTTCCTTAGACAGTTC
qPCR - Asnap	Reverse	CCATAGCTTGCTGGGCATTA
qPCR - Rop	Forward	GATGCCGAAGGAGAACGTATTA
qPCR - Rop	Reverse	GACAGGGTATACAGGAGGATGA

Table S4 ***Amplification efficiencies and melting temperatures of qRT-PCR primer pairs used in this study.***

Primer efficiencies were determined using serial cDNA dilutions. Each primer pair produced a single melting peak during melt-curve analysis, indicating specific amplification.

Primer name	Efficiency rate (E %)	Melting temperature (Tm)
qPCR - Rop	105.9	≈ 78.8 °C
qPCR - AchE	95	≈ 78.7 °C
qPCR - Asnap	99.6	≈ 78.2 °C
qPCR - 18srRNA	99	≈ 77 °C

Table S5 ***Total number of adult Halyomorpha halys insects used in each experimental cohort and treatment group.***

Independent injection series are indicated by the suffixes "-1", "-2", and "-3". Treatments included dsRNA targeting selected genes and nuclease-free water controls.

Treatment	Total treated-insects
dsRNA-ACHE - 1	16
dsRNA-ACHE - 2	8
dsRNA-ACHE - 3	7
dsRNA-ASNAP - 1	15
dsRNA-ASNAP - 2	4
dsRNA-ASNAP - 3	12
dsRNA-ROP - 1	15
dsRNA-ROP - 2	9
dsRNA-ROP - 3	10
dsRNA-TH - 1	17
dsRNA-SH - 1	13
NF-water - 1	16
NF-water - 2	9
NF-water - 3	9

Table S6 ***RNA concentration and spectrophotometric purity measurements of RNA samples used for cDNA synthesis and qRT-PCR analyses.***

RNA concentration (ng/µL) and absorbance ratios (260/280 and 260/230) were measured using UV/Vis spectrophotometry to assess RNA quality prior to reverse transcription.

Sample name	RNA concentration	260/280	260/230
dsROP inj. 1	966	2.08	2.46
dsROP inj. 2	903	2.05	2.35
dsROP inj. 3	963	2.05	2.35
dsROP inj. 4	998	2.05	2.41
dsROP inj. 5	1043	2.04	2.41
dsROP inj. 6	887	2.1	2.46
dsROP inj. 7	930	2.06	2.4
dsAchE inj. 1	932	2.06	2.42
dsAchE inj. 2	1118	1.94	2.13
dsAchE inj. 3	1014	2.06	2.4
dsAchE inj. 4	863	2.1	2.5
dsAchE inj. 5	1126	2.01	2.34
dsAchE inj. 6	1003	2.09	2.13
dsAchE inj. 7	1183	1.83	2.07
dsAsnap inj. 1	1096	2.01	2.39
dsAsnap inj. 2	1129	1.95	2.22
dsAsnap inj. 3	1045	2.09	2.4
Water inj. 1	1013	2.05	2.39
Water inj. 2	1100	2.11	2.44
Water inj. 3	548	2.08	2.54
Water inj. 4	729	2.09	2.47
Water inj. 5	1096	1.99	2.33
Water inj. 6	762	2.01	2.03
Water inj. 7	922	2.1	2.4

Table S7 ***RT-qPCR data used for relative transcript abundance analysis following dsRNA treatment in Halyomorpha halys.***

Individual biological replicates are shown for Rop, AChE, and Asnap, including ΔCt, ΔΔCt, and relative expression values calculated using the 2^−ΔΔCt method. Water-injected insects served as controls.

Treatment	Target gene	ΔCt	ΔΔCt	2^-(ΔΔCt)	Replicate	Batch
Water	Ras opposite	-0.75521	0.360499	0.778895	1	1
Water	Ras opposite	-1.26419	-0.14848	1.108401	2	1
Water	Ras opposite	-1.53005	-0.41434	1.332691	3	1
Water	Ras opposite	-1.79441	-0.6787	1.600693	4	1
Water	Ras opposite	-0.88613	0.229583	0.852881	5	2
Water	Ras opposite	0.176769	1.292482	0.408248	6	2
Water	Ras opposite	-1.75676	-0.64105	1.559459	7	2
dsROP	Ras opposite	0.233449	1.349161	0.39252	1	1
dsROP	Ras opposite	-0.41973	0.695987	0.617287	2	1
dsROP	Ras opposite	0.609103	1.724815	0.302537	3	1
dsROP	Ras opposite	-0.24711	0.868601	0.547678	4	1
dsROP	Ras opposite	0.303289	1.419001	0.373971	5	2
dsROP	Ras opposite	-0.49493	0.620782	0.650318	6	2
dsROP	Ras opposite	0.255641	1.371354	0.386528	7	2
Water	Acetylcholinesterase	3.230495	1.253721	0.419365	1	1
Water	Acetylcholinesterase	2.704083	0.727309	0.60403	2	1
Water	Acetylcholinesterase	0.336295	-1.64048	3.117693	3	1
Water	Acetylcholinesterase	1.289498	-0.68728	1.61024	4	1
Water	Acetylcholinesterase	1.687613	-0.28916	1.221929	5	2
Water	Acetylcholinesterase	3.491639	1.514866	0.349929	6	2
Water	Acetylcholinesterase	1.097793	-0.87898	1.839075	7	2
dsAchE	Acetylcholinesterase	3.792746	1.815973	0.284013	1	1
dsAchE	Acetylcholinesterase	4.054408	2.077634	0.236903	2	1
dsAchE	Acetylcholinesterase	1.523963	-0.45281	1.368704	3	1
dsAchE	Acetylcholinesterase	1.873397	-0.10338	1.074285	4	1
dsAchE	Acetylcholinesterase	1.868034	-0.10874	1.078286	5	2
dsAchE	Acetylcholinesterase	3.359154	1.38238	0.383585	6	2
dsAchE	Acetylcholinesterase	0.803105	-1.17367	2.255847	7	2
Water	Alpha-SNAP	-2.68775	-0.23568	1.17746	1	2
Water	Alpha-SNAP	-1.51337	0.938703	0.521702	2	2
Water	Alpha-SNAP	-3.1551	-0.70302	1.627914	3	2
dsAsnap	Alpha-SNAP	-2.58598	-0.13391	1.097264	1	2
dsAsnap	Alpha-SNAP	-3.52478	-1.07271	2.103376	2	2
dsAsnap	Alpha-SNAP	-2.81971	-0.36763	1.290235	3	2

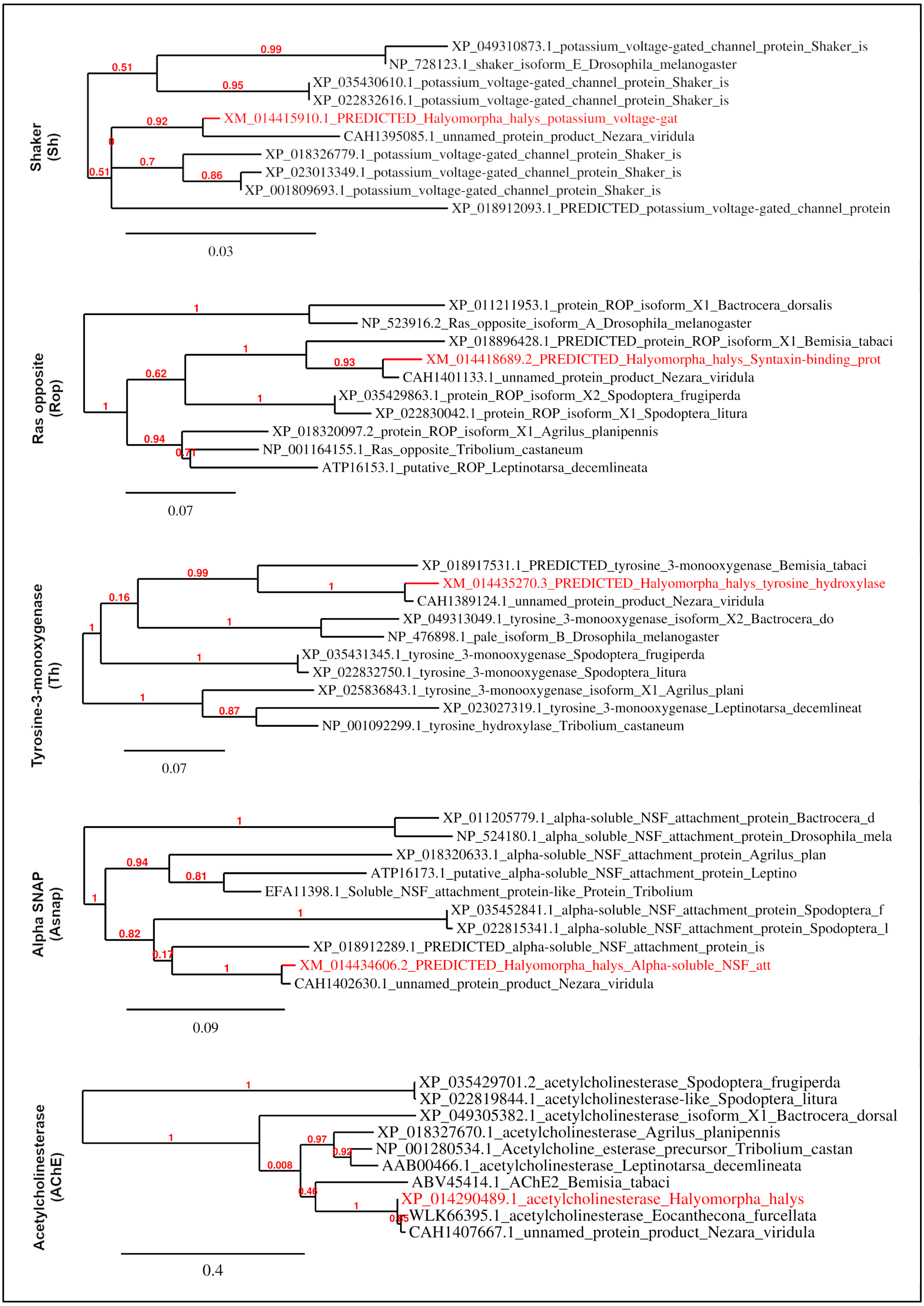

Figure S1 ***Maximum-likelihood phylogenetic analysis of candidate RNAi target proteins identified in Halyomorpha halys.***

Phylogenetic trees were generated using homologous protein sequences from representative insect species with the Phylogeny.fr platform. H. halys sequences identified in this study are highlighted in red. Branch values indicate aLRT support values, and scale bars represent amino acid substitutions per site.

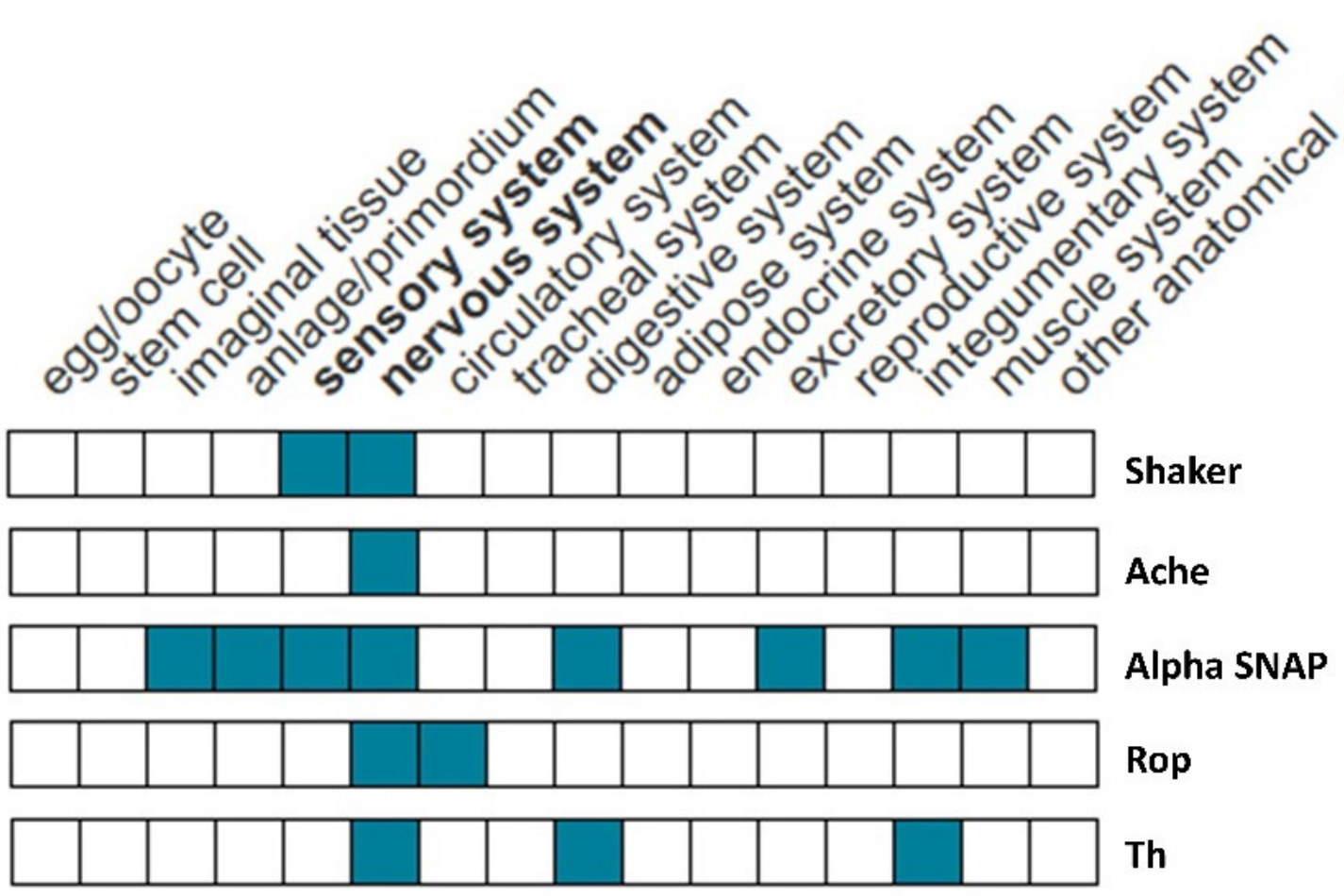

Figure S2 ***Tissue-specific expression summary of selected candidate genes in Drosophila melanogaster based on curated FlyBase expression data (FB2025_05).***

Colored tiles indicate anatomical systems in which RNA and/or protein expression evidence is available, whereas uncolored tiles indicate absence of curated expression data.

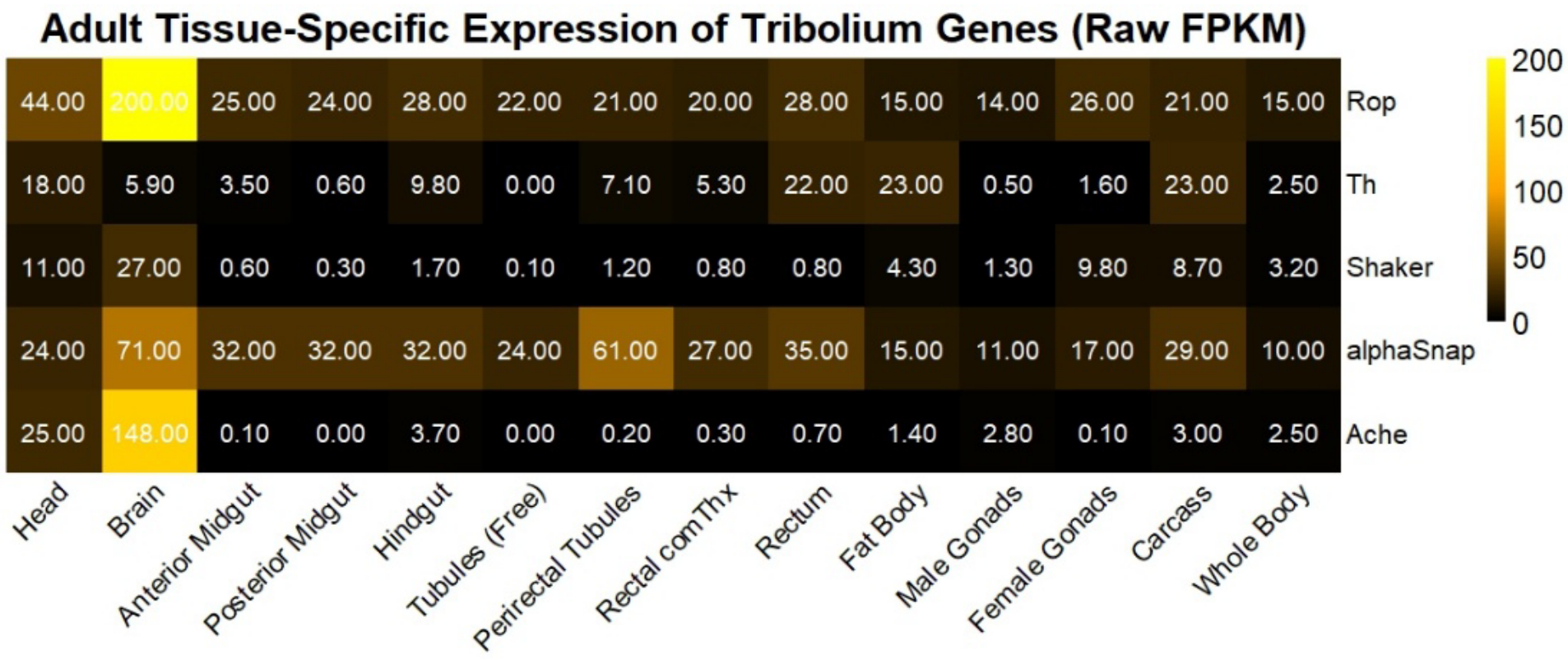

Figure S3 ***Adult tissue-specific expression patterns of selected candidate genes in Tribolium castaneum based on BeetleAtlas RNA-seq data.***

Expression levels are shown as raw FPKM values across dissected adult tissues, including neural and non-neural organs. Color intensity represents relative transcript abundance within each tissue. Asnap and Rop displayed broader tissue distribution and higher overall transcript abundance compared with AChE, Th, and Sh.

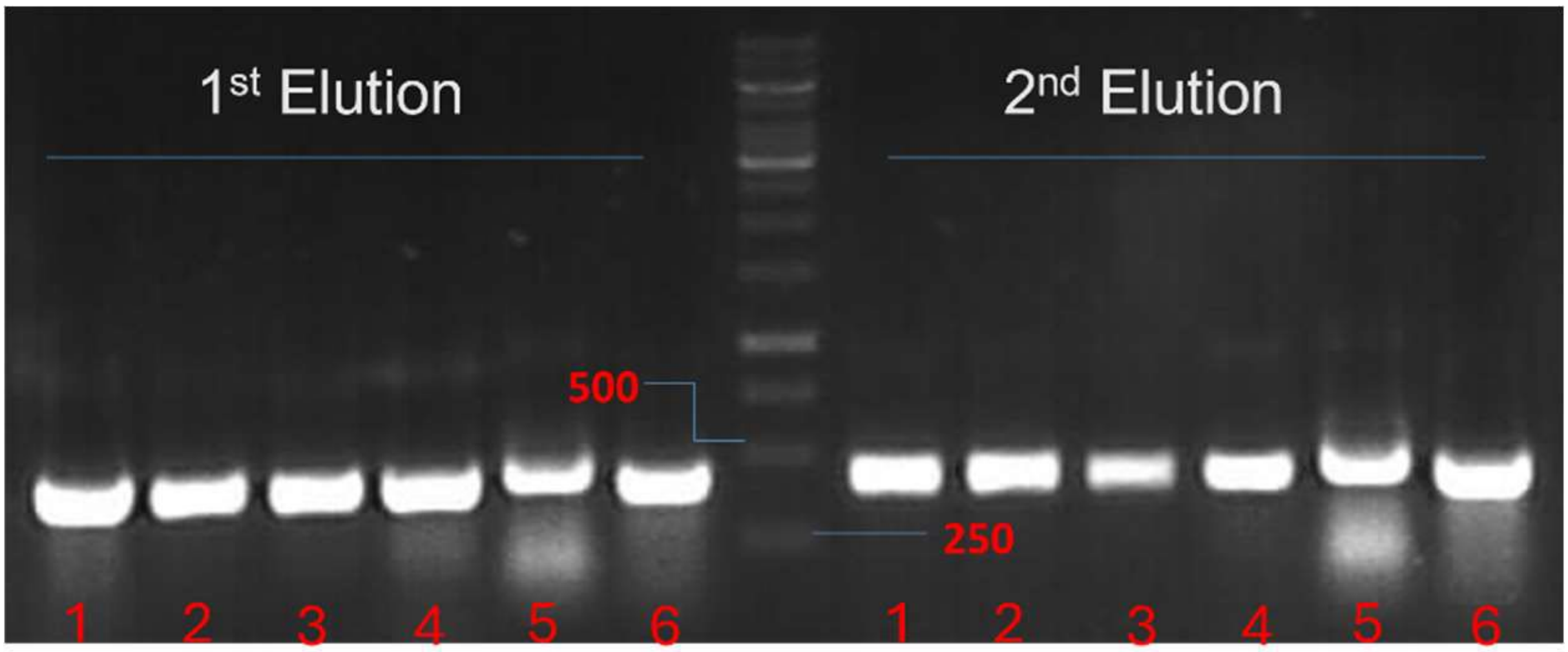

Figure S4 ***Agarose gel electrophoresis analysis of purified dsRNA products synthesized for RNAi experiments.***

Purified dsRNA targeting AChE (342 bp), Asnap (334 bp), Rop (340 bp), Sh (364 bp), and Th (334 bp) was analyzed on a 1% agarose gel following in vitro transcription and purification. Comparable band intensity across elution fractions indicates successful dsRNA synthesis and high RNA integrity. Lane assignments correspond to: 1, AChE; 2, Asnap; 3, Rop; 4, Sh; 5, Th.

References:

Ellango, R., Asokan, R., Chandra, G. S., Kumar, N. K. K., Mahmood, R., & Ramamurthy, V. V. (2018). Tyrosine Hydroxylase, a Potential Target for the RNAi-Mediated Management of Diamondback Moth (Lepidoptera: Plutellidae). *Florida Entomologist*, *101*(1), 1–5. https://doi.org/10.1653/024.101.0102

Knorr, E., Fishilevich, E., Tenbusch, L., Frey, M. L. F., Rangasamy, M., Billion, A., Worden, S. E., Gandra, P., Arora, K., Lo, W., Schulenberg, G., Valverde-Garcia, P., Vilcinskas, A., & Narva, K. E. (2018). Gene silencing in Tribolium castaneum as a tool for the targeted identification of candidate RNAi targets in crop pests. *Scientific Reports*, *8*(1), 2061. https://doi.org/10.1038/s41598-018-20416-y

Malik, H. J., Raza, A., Amin, I., Scheffler, J. A., Scheffler, B. E., Brown, J. K., & Mansoor, S. (2016). RNAi-mediated mortality of the whitefly through transgenic expression of double-stranded RNA homologous to acetylcholinesterase and ecdysone receptor in tobacco plants. *Scientific Reports*, *6*(1), 38469. https://doi.org/10.1038/srep38469

Mysore, K., Hapairai, L. K., Sun, L., Li, P., Wang, C.-W., Scheel, N. D., Lesnik, A., Igiede, J., Scheel, M. P., Wei, N., Severson, D. W., & Duman-Scheel, M. (2020). Characterization of a dual-action adulticidal and larvicidal interfering RNA pesticide targeting the Shaker gene of multiple disease vector mosquitoes. *PLOS Neglected Tropical Diseases*, *14*(7), e0008479. https://doi.org/10.1371/journal.pntd.0008479

Mysore, K., Sun, L., Hapairai, L. K., Wang, C.-W., Roethele, J. B., Igiede, J., Scheel, M. P., Scheel, N. D., Li, P., Wei, N., Severson, D. W., & Duman-Scheel, M. (2021). A Broad-Based Mosquito Yeast Interfering RNA Pesticide Targeting Rbfox1 Represses Notch Signaling and Kills Both Larvae and Adult Mosquitoes. *Pathogens*, *10*(10), 1251. https://doi.org/10.3390/pathogens10101251

Qu, X., Wang, S., Lin, G., Li, M., Shen, J., & Wang, D. (2022). The Synergistic Effect of Thiamethoxam and Synapsin dsRNA Targets Neurotransmission to Induce Mortality in Aphis gossypii. *International Journal of Molecular Sciences*, *23*(16), 9388. https://doi.org/10.3390/ijms23169388

Rival, T., Soustelle, L., Strambi, C., Besson, M.-T., Iché, M., & Birman, S. (2004). Decreasing Glutamate Buffering Capacity Triggers Oxidative Stress and Neuropil Degeneration in the Drosophila Brain. *Current Biology*, *14*(7), 599–605. https://doi.org/10.1016/j.cub.2004.03.039

Rodrigues, T. B., Duan, J. J., Palli, S. R., & Rieske, L. K. (2018). Identification of highly effective target genes for RNAi-mediated control of emerald ash borer, Agrilus planipennis. *Scientific Reports*, *8*(1), 5020. https://doi.org/10.1038/s41598-018-23216-6

Tariq, K., Ali, A., Davies, T. G. E., Naz, E., Naz, L., Sohail, S., Hou, M., & Ullah, F. (2019). RNA interference-mediated knockdown of voltage-gated sodium channel (MpNav) gene causes mortality in peach-potato aphid, Myzus persicae. *Scientific Reports*, *9*(1), 5291. https://doi.org/10.1038/s41598-019-41832-8

Ulrich, J., Dao, V. A., Majumdar, U., Schmitt-Engel, C., Schwirz, J., Schultheis, D., Ströhlein, N., Troelenberg, N., Grossmann, D., Richter, T., Dönitz, J., Gerischer, L., Leboulle, G., Vilcinskas,

A., Stanke, M., & Bucher, G. (2015). Large scale RNAi screen in Tribolium reveals novel target genes for pest control and the proteasome as prime target. *BMC Genomics*, *16*(1), 674. https://doi.org/10.1186/s12864-015-1880-y

Zhang, L., Cheng, X., Tao, S., Peng, L., Zhu, Z., & Bao, Y. (2022). Neuronal calcium sensor 2 is key to moulting and oocyte development in the brown planthopper, *Nilaparvata lugens*. *Insect Molecular Biology*, *31*(6), 722–733. https://doi.org/10.1111/imb.12799
